# Application of Cumulative Threshold Approaches to Tissue Iron Measurement on Histochemical or Synchrotron X‑ray Fluorescence Platforms

**DOI:** 10.1021/cbmi.5c00230

**Published:** 2026-02-16

**Authors:** Chan-An Lin, Elvis Acquah, Jake Brooks, Joanna F. Collingwood, Daniel M. Johnstone, Adrienne E. Milward, Rebecca J. Hood

**Affiliations:** † School of Medical, Indigenous and Health Sciences, Faculty of Science, Medicine and Health, 8691 University of Wollongong, Wollongong, NSW 2522, Australia; ∇ School of Medical, Indigenous and Health Science, Molecular Horizons, University of Wollongong, Wollongong, NSW 2522, Australia; ‡ 172842 University of Utah, Department of Pathology, Salt Lake City, Utah 84108, United States; § 2707 University of Warwick, School of Engineering, Coventry, England CV4 7AL, U.K.; ∥ University of Newcastle, School of Biomedical Sciences and Pharmacy, Callaghan, NSW 2308, Australia; ⊥ The Florey Institute of Neuroscience and Mental Health, University of Melbourne, Parkville, VIC 3052, Australia; # University of Adelaide, School of Biomedicine, Adelaide, SA 5005, Australia

**Keywords:** cumulative threshold analysis, iron, image analysis, histochemistry, synchrotron X-ray fluorescence

## Abstract

Digitized imaging of the spatial distribution of a targeted metal over a given area converts a continuous spectrum of data into a set of discrete pixels of intensities that ideally correlate strongly with the areal density map. This should be independent of the metal imaging modality, allowing semiquantitative analysis. In practice, correlation strength may vary between data sets obtained by different modalities. One enduring problem for all modalities is selecting a cutoff threshold intensity appropriately distinguishing the true signal from background noise. This may entail subjective choices between “conservative” thresholds prioritizing specificity over sensitivity (i.e., true positives despite possible false negatives) and “discovery-driven” thresholds prioritizing identification of putative effects for subsequent validation (i.e., true negatives despite possible false positives). Computerized data processing may help make this more objective by comparing outcomes for the set of all possible threshold values, termed cumulative threshold analysis. We address pitfalls in performing valid cumulative threshold analysis using ImageJ/Fiji for relative quantification of brain iron in mice with normal or genetically elevated iron, assessed by classical histochemistry or synchrotron X-ray fluorescence. In addition to pitfalls in choosing settings for generating analysis histograms, these include data loss with conversion between bit depths and selecting both appropriate *X*-axis directionality and image display range minima and maxima for analyzing multiple images. If these factors are handled appropriately, cumulative thresholding provides a powerful approach for more objective analysis of biometal imaging. This has important applications for metal imaging in research and clinical settings.

## Graphical abstract

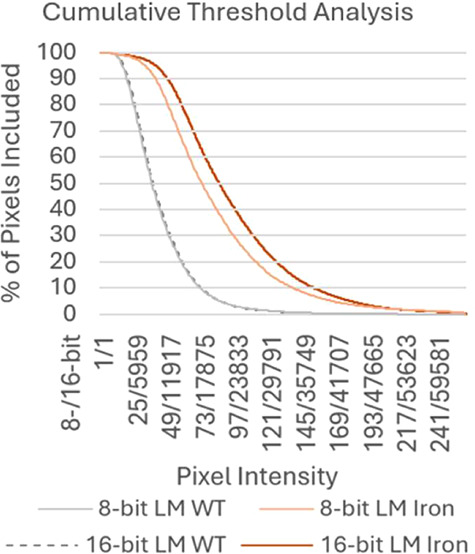

## Introduction

Image analysis has been used for relative or absolute quantification of image properties of interest, such as staining intensity.^1–3^ Due to complex matrix effects and other factors, current techniques for imaging biological iron, including synchrotron X-ray fluorescence (SXRF) as well as histochemical staining, do not necessarily provide accurate, absolute quantitative measurements of substances of interest but can still give useful relative quantitative comparisons of different samples. Appropriate selection of the signal from background noise is important for both absolute and relative quantification. Our intention here is to investigate pitfalls in this process for any image quantification, absolute or relative.

“Thresholding” is a common technique that attempts to distinguish noise from signal when analyzing images acquired from standard light, epi-fluorescent, confocal, or other kinds of microscopes, subsequently imported into image analysis programs.^3,4^ Determining an appropriate threshold for a map or image is often a subjective process involving the selection of a specific pixel intensity (PI) that ideally captures the maximum amount of labeled material of interest while filtering out background noise. Pixels detected within the intensity range are then quantified for comparative analysis of different groups of mapped data images.^5^

One limitation of this method is its reliance on arbitrarily chosen intensity thresholds, often only entailing a single cutoff point.^6,7^ These are usually picked by researchers adjusting cutoff values to meet nonstandardized subjective criteria. The selected value is often then applied to the remainder of the data set, which may contain images with different signal and noise distributions, or other arbitrarily selected thresholds are chosen for comparison groups. The lack of a consistent way to determine PI threshold values may compromise or weaken the reliability of the results.^3^ How thresholds are set is not stated in many papers, making reliability difficult, if not impossible, to assess.

Here, we consider potential pitfalls in threshold selection using examples from ImageJ/Fiji, widely used for image analysis in the bioimaging community and referred to here as ImageJ. Because ImageJ simplifies many complex operations for users, nonexperts may not understand or be aware of the underlying data manipulations and information loss which can be involved. We also look at how various settings can influence how analyses are performed in unexpected ways that can substantially affect the outcomes.

Digitized images generated using microscopy involve the transformation of continuous light energy signals captured by a sensor or detector into categorical binary classifications (e.g., white/black, 0/1). This necessarily involves information loss, with the extent of the loss depending in part on how the data are grouped and organized and specifically the bit depth and type used to represent the data. More information is lost with lower bit depths, notably the widely used 8-bit depth. This divides the pixel intensity range between absolute black and absolute white into 2^8^ intensity intervals or “bins,” i.e., 256, approximately a quarter of the number a human eye can resolve.^8^ With regard to pixel intensity, higher bit formats such as 16-bit or 32-bit have more intervals, e.g., 65,535 or 4,294,967,296 respectively, providing higher radiometric resolution. Hence, the 8-bit format effectively collapses *thousands* of intensities into each single bin relative to 16-bit and *thousands of millions* into a single bin relative to 32-bit. The numbers of intensities able to be represented with each format are given in Table 1.

**Table 1:**
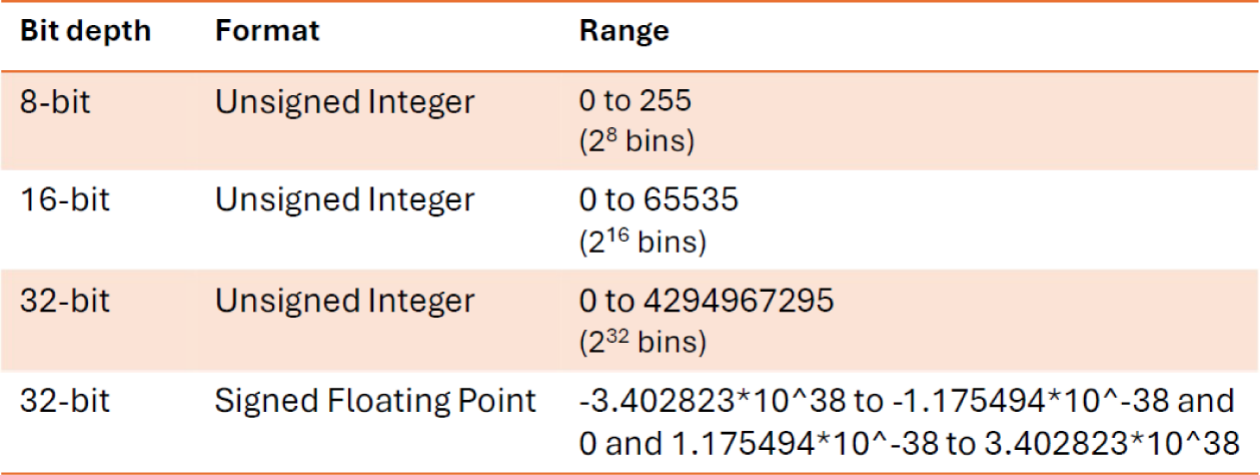
Bin Numbers for Different Bit Formats and Types (IEEE Std 754-2019)

For example, when converting between bit depths, it is important to understand the bit types that the data analysis program uses and how data are modified or lost, particularly when converting images to 8-bit, as is widely done. ImageJ uses unsigned integers for 8- and 16-bit depths and floating point for 32-bit depths. According to current international Institute of Electrical and Electronics Engineers (IEEE) 754 standards, 32-bit coding uses 24 bits to encode significant binary digits and 8 bits for other information (IEEE Std 754-2019).

Selecting thresholds to delineate signal from noise also necessarily entails information loss. The use of a single threshold to analyze a set of images assumes that differences between sample groups across the spectrum are constant.^3^ This can lead to inadvertently overlooking possible differences between groups or threshold manipulation to exaggerate or reduce actual group differences.^6^

Modern image analysis programs have tools to assist in reducing potential issues of subjective selection of a threshold cutoff level. This includes functions or macros that can automatically process the areal density curve over the full range of the available data in the format used. The areal density curve represents the distribution of data points across a continuous interval or area, revealing where the values are concentrated. Density curve processing can be done in various ways for easier comparison between different PI ranges to allow more objective threshold selection.^4^ FIJI/ImageJ version 2.9.0/1.54 currently contains 17 different autothreshold algorithms, including Otsu Thresholding,^9^ Huang Fuzziness,^10^ Maximum Entropy threshold setting approaches that select thresholds that aim to optimize the information obtained from comparisons of signal to background,^11^ and others.^10,12,13^ For example, in the unbiased automatic threshold selection technique proposed by Otsu, the data distribution is assumed to be bimodal, composed of separate background (“valley”) and signal intensity distributions.^9^ This method is more appropriate than arbitrary subjective approaches of selecting threshold values but is often not suitable for biological data, which can instead consist of mixture distributions comprising separate signal and noise subdistributions that are often complex in nature.

Considering the full range of data from image data sets, or at least a wider range, when selecting threshold cutoffs can lower the chance of missing important differences or overinterpreting nonrepresentative differences and reduce the opportunities for biased modification of threshold points to affect outcomes.^14^ Johnson and Walker (2015) suggested a cumulative threshold approach can be used to improve threshold selection strategies.^3^ Their method, which utilizes 8-bit formatting, makes use of information from the entire data spectrum available for this format to empirically determine an acceptable range of threshold pixel intensities for data analysis. This facilitates more accurate quantification than traditional approaches relying on single arbitrarily picked PIs, although still requiring user selection of the minimum and maximum limits of the threshold range.

Here, we investigate the application of this method to images obtained from enhanced histochemical analyses of brain iron in control mice and mice with genetic mutations related to the human iron disorder hemochromatosis, which exhibit strongly elevated iron loading (Figure 1). The chemical used to enhance iron visualization, 3,3′-diaminobenzidine (DAB), is the same as that used by Johnson and Walker, although the experimental context and objects of interest differ. For comparison, we also analyzed synchrotron X-ray fluorescence (SXRF) images of iron in a small region of the brain with high iron content, generating images with high resolution but few pixels compared with the microscopy images of whole brain sections. A schematic exemplifying how signal intensities in the SXRF images were determined is shown in Figure S1, Supporting Information.

**Figure 1:**
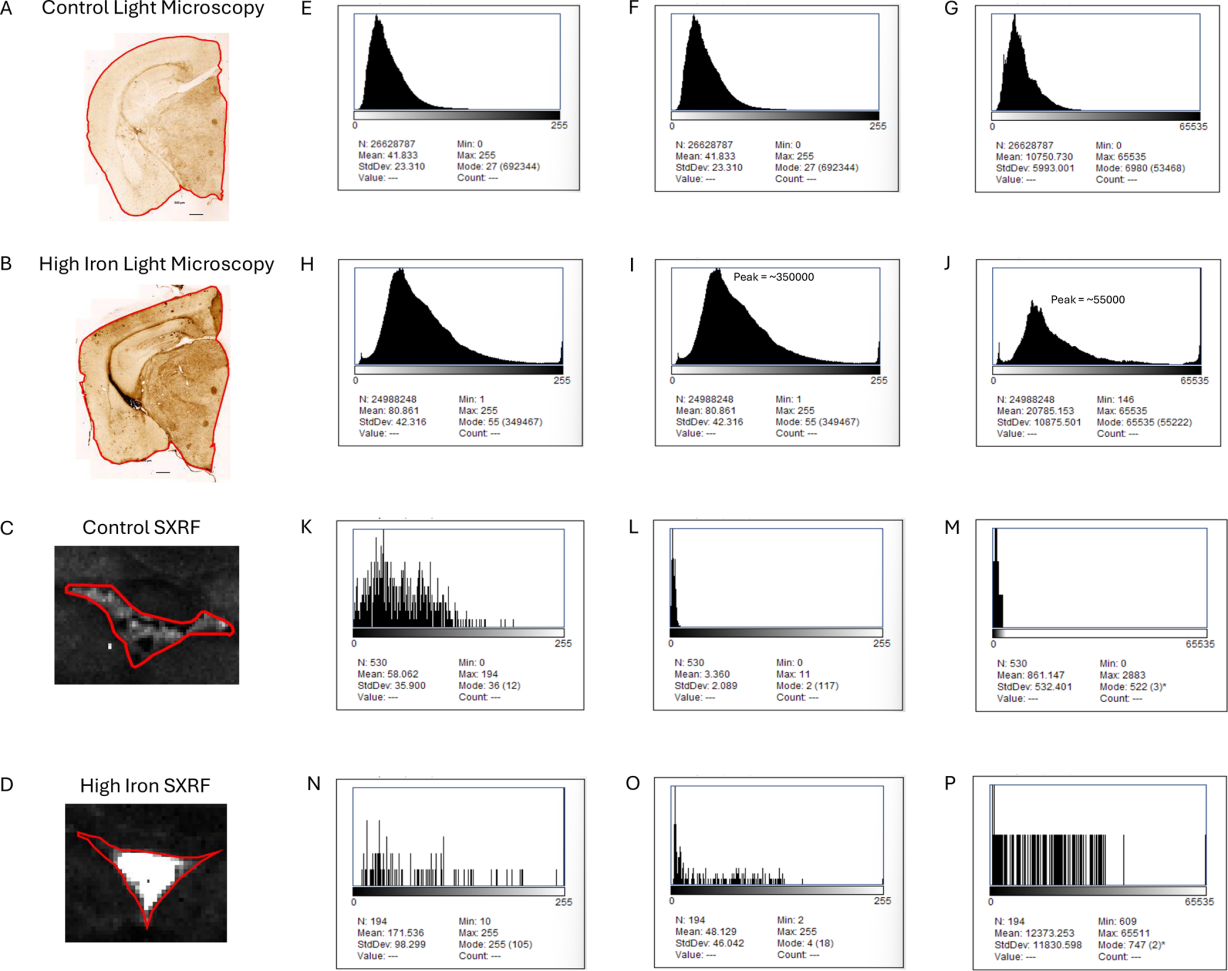
Representative images of light microscopy (A, B) and SXRF (C, D) used for analysis histograms. The first histogram shown for each image (E, H, K, and N) has been obtained at 8-bits using the ImageJ *Brightness/Contrast* and *Histogram* default settings, where the two numbers at the ends of each histogram are the minimum and maximum PI values of the data range. The second (F, I, L, and O) and third (G, J, M, and P) sets of histograms show, for 8- and 16-bit formats, respectively, the histograms obtained when the display range for each of the two images for each platform is set before conversion to these lower bit depths to a minimum of 0 and a maximum corresponding to the maximum PI of those two images.

Importantly, we have explored the use of two widely used programs, ImageJ and Excel, for performing cumulative threshold analysis. We identify several issues that can arise when applying cumulative thresholding approaches using ImageJ and ways in which these can be minimized. With these caveats, cumulative threshold analysis offers important benefits for transparent, unbiased analyses of iron and other metals across diverse platforms.

## Methods

### Ethics

Tissue samples for histology were obtained by Dr. Dan Johnstone and Dr. Elvis Acquah from mice held at the University of Sydney under protocols approved by the Animal Ethics Committee of the University of Sydney (protocol number 2017/1188), in accordance with the guidelines of the National Health and Medical Research Council (NHMRC) of Australia.

### Animals

The examples of mouse brain images presented in this paper were acquired from *Hfe* ^*–/–*^ *xTfr2* ^*mut*^ mice with elevated brain iron loading (High Iron model) and age- and sex-matched wild-type control mice, all at 9 months of age. The High Iron model was generated by crossing mice with homologous recombinant deletion of the *Hfe* gene (*Hfe* ^*–/–*^)^15^ and mice with the Y245X nonsense mutation in the *Tfr2* gene (*Tfr2* ^*mut*^).^16^ These mice were maintained on an AKR genetic background^15,16^ which has stronger iron-loading capability compared to other strains studied.^17,18^ The APP/PS1 transgenic mice (Control Aβ model) express mutant human amyloid precursor protein (Mo/HuAPP695swe) and presenilin-1 (PS1-ΔE9) transgenes on a mixed C3HxC57BL/6 background (Jackson Laboratories strain stock number 004462). The APP/PS1x *Hfe* ^*–/–*^ *xTfr2* ^*mut*^ mice (High Iron Aβ model) were created by crossing APP/PS1 transgenic mice with *Hfe* ^*–/–*^ *xTfr2* ^*mut*^ mice, then backcrossing the offspring onto the AKR background for 10 generations. These mice are used to illustrate selection of iron-containing entities (amyloid plaques, fibrous structures) and not for comparisons involving Aβ so are also referred to as Control and High Iron respectively.

### Tissue Fixation and Sectioning for Histochemistry

Mice were sacrificed at ages of interest by intraperitoneal injection of 60 mg/kg sodium pentobarbitone (Lethabarb, Birvac Australia Pty Ltd.), followed by cardiac puncture and transcardial perfusion with paraformaldehyde (PFA; Sigma-Aldrich), 4% in 1× phosphate-buffered saline (PBS) for histological studies. Following the perfusion, the tissue was stored in 4% paraformaldehyde (Sigma-Aldrich) at 4 °C overnight, then washed with PBS and transferred into 0.1% sodium azide (MERCK) in 1× PBS (PBS+azide; pH 7.4) the next day, and stored at 4 °C until required. Brains were placed into a coronal or longitudinal mouse brain slicer matrix (Zivic Instruments) and sliced into blocks by inserting razor blades into the 5 slice channels and then stored in PBS+azide at 4 °C. Brain blocks were cryoprotected in sucrose to prevent ice crystal artifacts from forming. Cryomolds were stored at −20 °C until sectioning. The tissues were then sectioned on the cryostat, and 25 μm thick sections were collected onto poly-l-lysine hydrobromide-coated slides for histological staining as below.

### Histological Staining and Imaging of Brain Iron

Histochemical labeling of iron was performed using DAB-enhanced Perls’ stain for ferric (Fe^3+^) iron,^19^ which also detects ferrous (Fe^2+^) iron which converts rapidly to ferric iron. Tissue sections were transferred to 1% potassium ferrocyanide (AnalaR; pH < 1) in distilled water (ddH_2_O), incubated at room temperature for 30 min, and then washed three times for two min each round in ddH_2_O. Sections were then incubated in 0.01 M sodium azide and 0.3% hydrogen peroxide (H_2_O_2_; Sigma-Aldrich) in methanol at room temperature for 1 h and then washed three times for two min each round in PBS (0.1 M, pH 7.4). Slides were then incubated for 30 min in DAB (MP Biomedical) solution (0.025 g DAB and 16.7 μL of 30% H_2_O_2_ added to 100 mL 0.1 M PBS; pH 7.4) and washed with ddH_2_O for three times with 2 min each round. Sections were dehydrated after staining using a graded series of ethanol washes (50%, 70%, 90%, 2 × 100%; two min each) and then cleared twice in 100% xylene for five min each. Coverslips were applied with Depex mounting medium (BDH Chemicals), and slides were imaged after drying overnight on the ZEISS Axio Scan.Z1 Slide Scanner system (University of Sydney).

Here, we analyzed histochemically stained sections from one High Iron mouse aged 9 months and one matched wild-type control mouse (Figure 1A, B).

### Tissue Fixation and Image Acquisition for SXRF

For comparison, we also analyzed label-free maps of iron distribution collected using SXRF of the fourth ventricle region from another High Iron mouse aged 9 months and matched wild-type control mouse (Figure 1C, D). Mice were perfused with 0.1 M sodium cacodylate, brains snap-frozen, and 30 μm coronal cryosections mounted on quartz slides. SXRF mapping was performed at the Swiss Light Source by using an incident beam energy of 10.5 keV and a minimum beam diameter of 8 μm. SXRF maps were fitted and normalized to incident beam *I*_0_ using PyMCA software. Output images were saved as TIFF files to preserve the numerical values for each pixel.

### Comparisons of Different Threshold Choices for Light Microscopy Images

The *Threshold* function in ImageJ at pixel intensities of 25, 50, 75, 100, and 125 (corresponding to 10%, 20%, 30%, 40%, and 50% of the total intensity range) was applied to four randomly selected regions of the same size from each of the control and High Iron full hemisphere images. The *Create Mask* function was then used to generate 8-bit binary images where pixels above the threshold were replaced by white with a PI of 255 and pixels below the threshold were replaced by black with a PI of 0 (not shown). These were used to create the red masked images that highlighted all pixels above each threshold (Figure 2). Alternatively, inverse mask (stencil) images that do not obscure the signal of interest were created by overlaying the inverse binary images to remove all pixels below the threshold and retain all details of signal pixels (Figures 2, 8, and 9).

### Cumulative Threshold Analysis

Images were converted to 8-bit or 16-bit grayscale image format in ImageJ, and effects of different conversion parameters (*Weighted RGB*, platform-related *X*-axis inversion, *Brightness/Contrast* settings) were investigated as detailed below (Results). Histograms were generated to visualize and compare data distributions in each bit format.

A running total of the cumulative count of the number of pixels excluded at each different threshold intensity was obtained by recording the number of pixels excluded at that intensity and adding this value to the cumulative count of pixels excluded at all lower threshold intensity settings until all pixel threshold intensities in the range of each bit depth (8- or 16-bit) were assessed. For comparisons of multiple images, the output values were then normalized by converting the pixel count to a percentage of the total pixel number within the selected region of interest (ROI). Plotting the sum of the normalized values of the pixel counts below the individual threshold at each PI generated a cumulative threshold graph for each image, as detailed below.

## Results

### Sample Images Used in Analyses

The examples of mouse brain images presented in this article were acquired from representative Iron model mice and representative control mice at 9 months of age. Representative images of coronal sections of brain hemispheres from control mice with normal brain iron loading and Iron mice (high brain iron) labeled with DAB + Perls’ histochemical stain for ferric iron (Fe^3+^) are shown in Figure 1A, B (Introduction). Images of the fourth brain ventricle in coronal sections from different control and Iron mice obtained by label-free SXRF were also included for analysis. This platform is likely to have different levels of resolution and sensitivity compared with traditional histochemical staining. Representative images are shown in Figure 1C, D.

### Using “Masking” or “Stencils” to Visualize Effects of Threshold Setting

To illustrate how threshold choice can affect image analysis outcomes, we compared the effects of analyzing randomly selected areas from the light microscopy control and High Iron hemisphere images using different threshold PIs to determine the proportion of pixels included as a signal (Figure 2A). We first applied the approach of Johnson and Walker (2015), which used the ImageJ masking function to highlight the pixels being included at each of the thresholds selected.^3^ However, we found this visualization approach counterintuitive as it obscures the areas of interest (i.e., signal), so an alternative reverse masking (“stencil”) approach was applied over the background areas instead to highlight the information retained (Figure 2B). Reverse masking has been used for the remainder of the paper.

The results suggest that using a single threshold determined from an individual image may not always be appropriate for other images in the analyses. Furthermore, even using a threshold determined from a subset of images, e.g., control images, may not be appropriate for all remaining images. Analyzing changes in the amount of signal determined by five different thresholds (Figure 2C) shows that group differences can change significantly between two thresholds even between similar looking selections (e.g., PI 100, 125).

**Figure 2:**
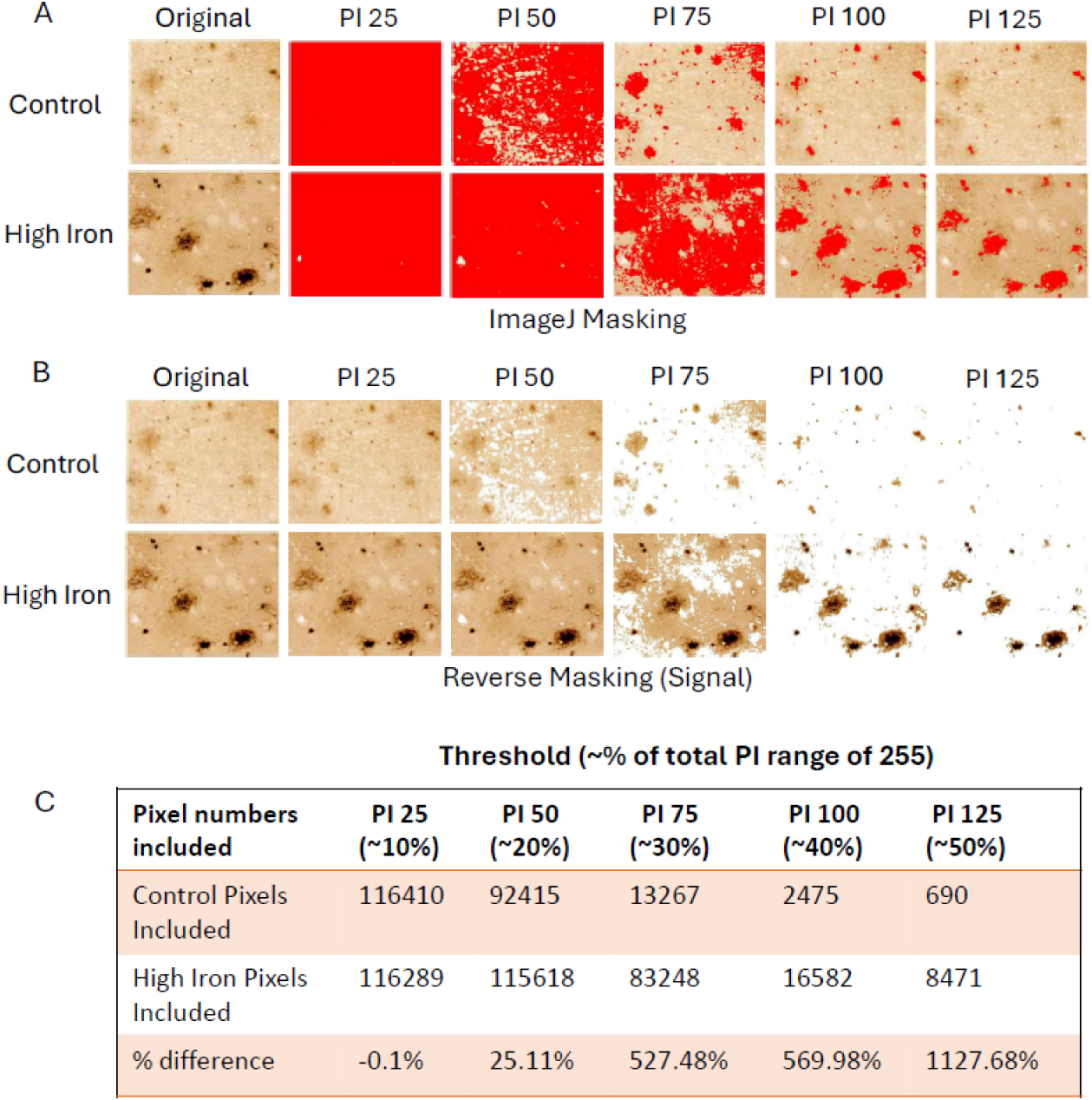
Masks, reverse masks (stencils), and effects of different threshold settings on the assessment of total or object-specific histochemical iron staining for light microscopy images of control and high iron mouse brains. The figure shows comparisons of pixel counts in areas A. retained (red mask) or B. excluded (white reverse mask, i.e., stencil) at different pixel intensity thresholds representing ∼10% to 50% of the total pixel intensity range. Iron-containing Alzheimer’s disease amyloid plaques, visible as large, dark patches, are excluded from the control (normal) iron image when high thresholds are used (PI 100, 125). C. Effects of different threshold choices on the relative quantification of histochemical iron staining and apparent magnitudes of differences between samples.

### Conversion of 24-Bit RGB Color Format to Grayscale for Image Analysis

The 24-bit Red Green Blue (RGB) color format consists of three separate 8-bit color channels for red, green, and blue. As illustrated in Figure 3, how colored photomicrograph images are converted from RGB to any grayscale format is determined by the *Conversion Option* setting in the *Option* menu of *Edit*. The default setting (*Weighted RGB* unselected) averages the values in each of the color channels. Alternatively, if the *Weighted RGB* option is selected, the National Television System Committee (NTSC) formula is applied (grayscale value = 0.299 × red value + 0.587 × green value + 0.114 × blue value).^20^ ImageJ allocates lower PIs to darker pixels and higher PIs to brighter pixels. This is inappropriate for platforms where signal is darker, e.g. histochemistry, and can be reversed using *Invert* in *Edit*. This corrects the PI values but generates a photographic negative version of the image (see Figure 3). Specific colors are assigned to each PI value by the Look-Up Table (LUT) and this can be corrected by the *Invert LUTs* command in *Color* in *Image*.

**Figure 3:**
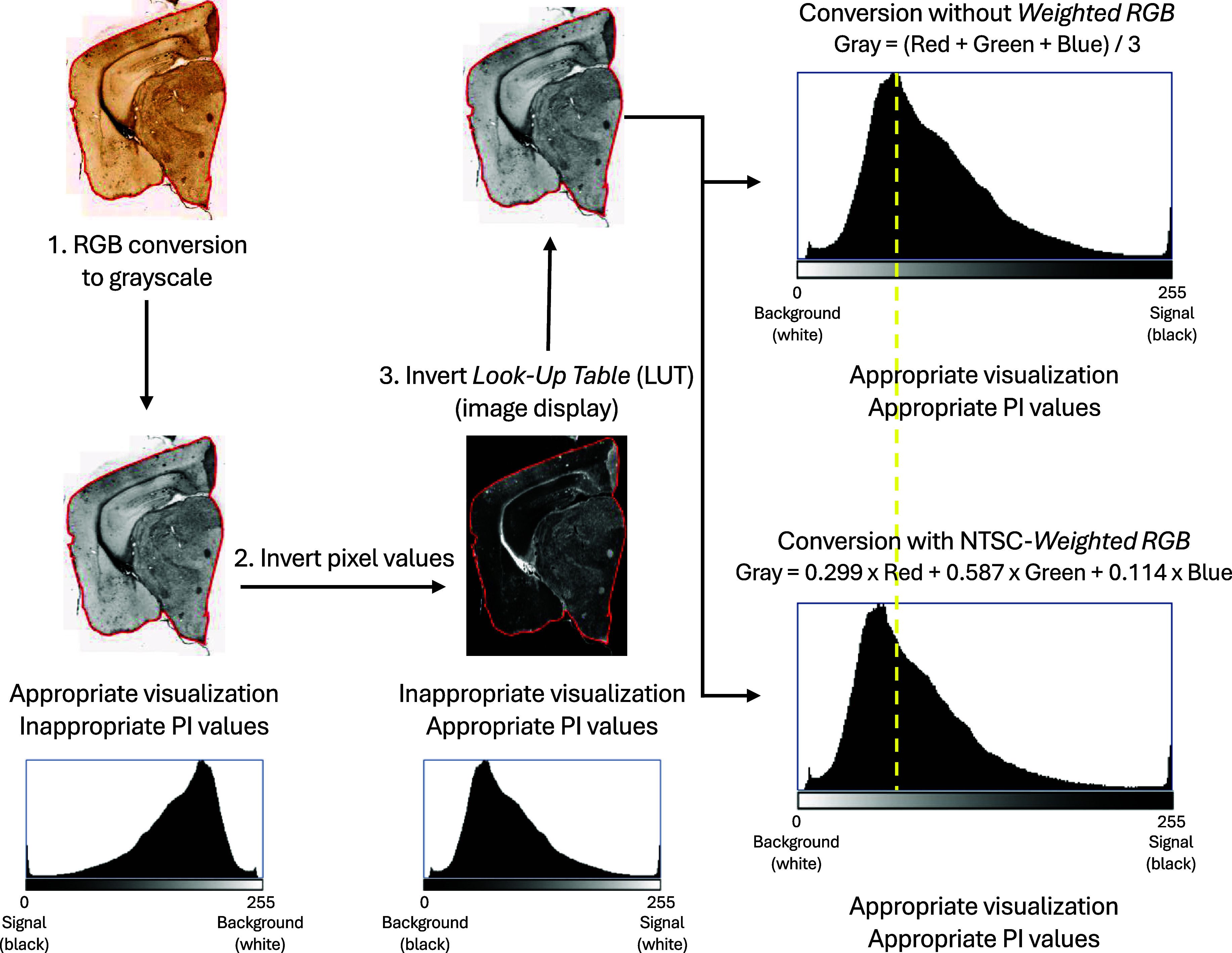
Use of different *Conversion Option* settings for RGB to grayscale conversion. The example features a large red component and shows a distribution shift toward higher intensities from averaging the color channels when*Weighted RGB* is unselected compared to when *Weighted RGB* is selected and using the NTSC formula.^20^

Depending on the final grayscale bit format to which the data are converted (selected from the *Type* menu), the resulting outputs may either be decimal numbers (for 32-bit) or integers after rounding of decimals (for 8- and 16-bit), as detailed in Table 1 (Introduction). Direct conversion to 8-bit is widely used for ease of analysis, but higher bit formats may help in differentiating pixels that have different color channel combinations but appear similar in intensity after grayscale conversion. However, it is important to recognize that the extra decimal information after converting to grayscale may not be an exact representation of pixels in the original colored image.

The grayscale range does not automatically get rescaled for the format to which it is converted, so converting from RGB directly to 16-bit will result in a range of 0–255. Converting RGB first to 32-bit and then to 16-bit retains some of the information represented by the decimal values and allows the range to linearly scale to 0–65,535.

It is essential to recognize that, for all analyses discussed in this paper, converting image data to a lower bit depth involves an irreversible loss of information for the image being analyzed, which cannot be undone by reverting to a higher bit depth. For this reason, it is essential to always use a working copy of any image being analyzed and not the original image.

### Comparisons of Cumulative Threshold Spectra

We next compared cumulative threshold spectra for light microscopy and SXRF images of pixels retained after picking threshold values at each PI (Figures 4 and 5). As further explained below, in constructing plots of these spectra and performing analyses, it is important to recognize that these two platforms differ with regard to the appropriate directionality of the *X*-axis scale relative to the ImageJ default and that if this is not realized, it can invalidate analyses.

**Figure 4:**
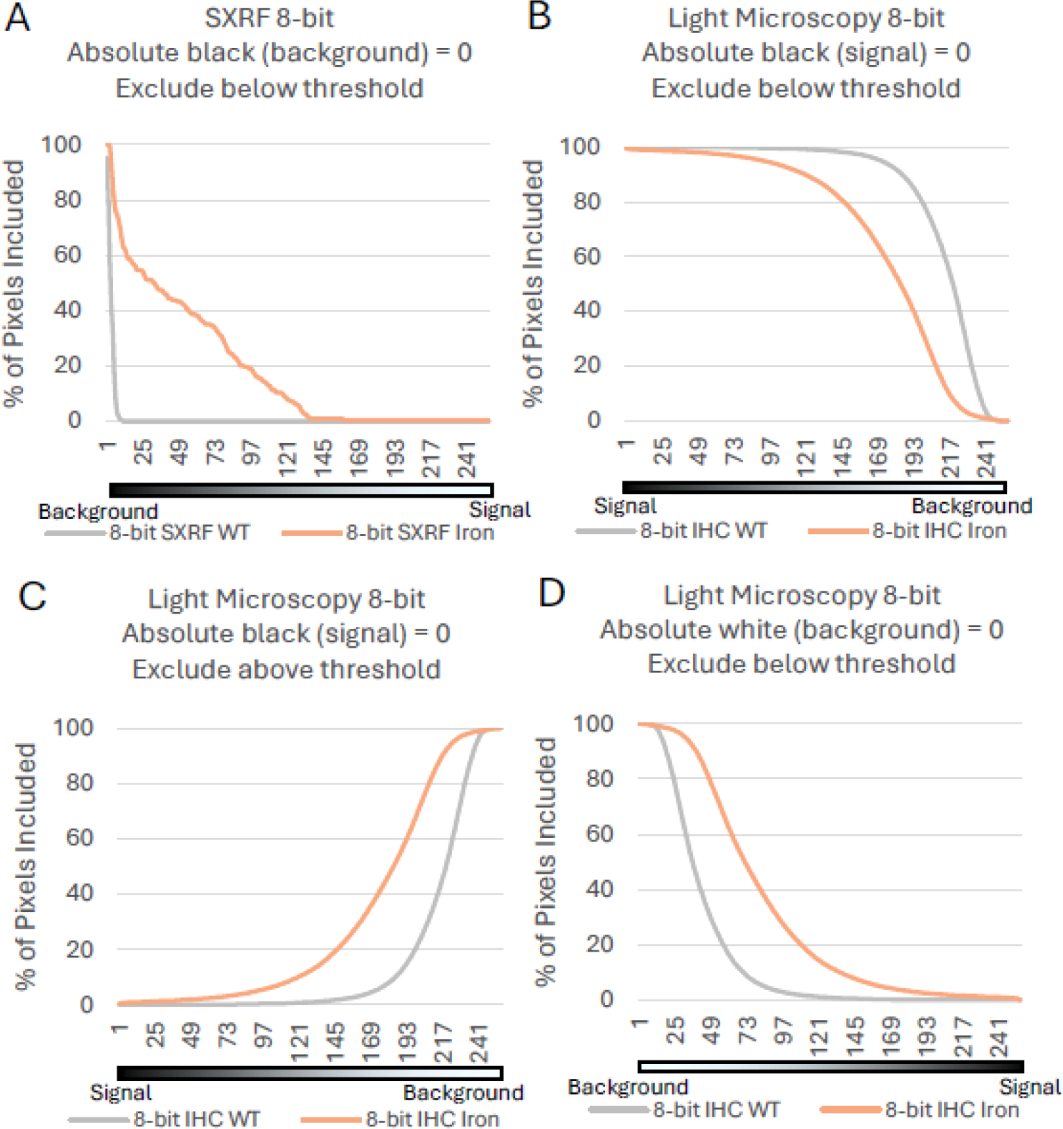
Cumulative threshold spectra for SXRF (A) and light microscopy (B-D) images of pixels retained after picking threshold values at each PI. (A, D) Pixels excluded below the threshold when the *X*-axis is set with signal values higher than background—correct setting and valid outcome. (B) Pixels excluded below the threshold when *X*-axis set with background values higher than signal—incorrect setting and invalid outcome. (C) Pixels excluded above the threshold when *X*-axis set with background values higher than signal—compensates for the incorrect setting, giving valid outcome. All graphs shown for the 8-bit format (256 bins).

**Figure 5:**
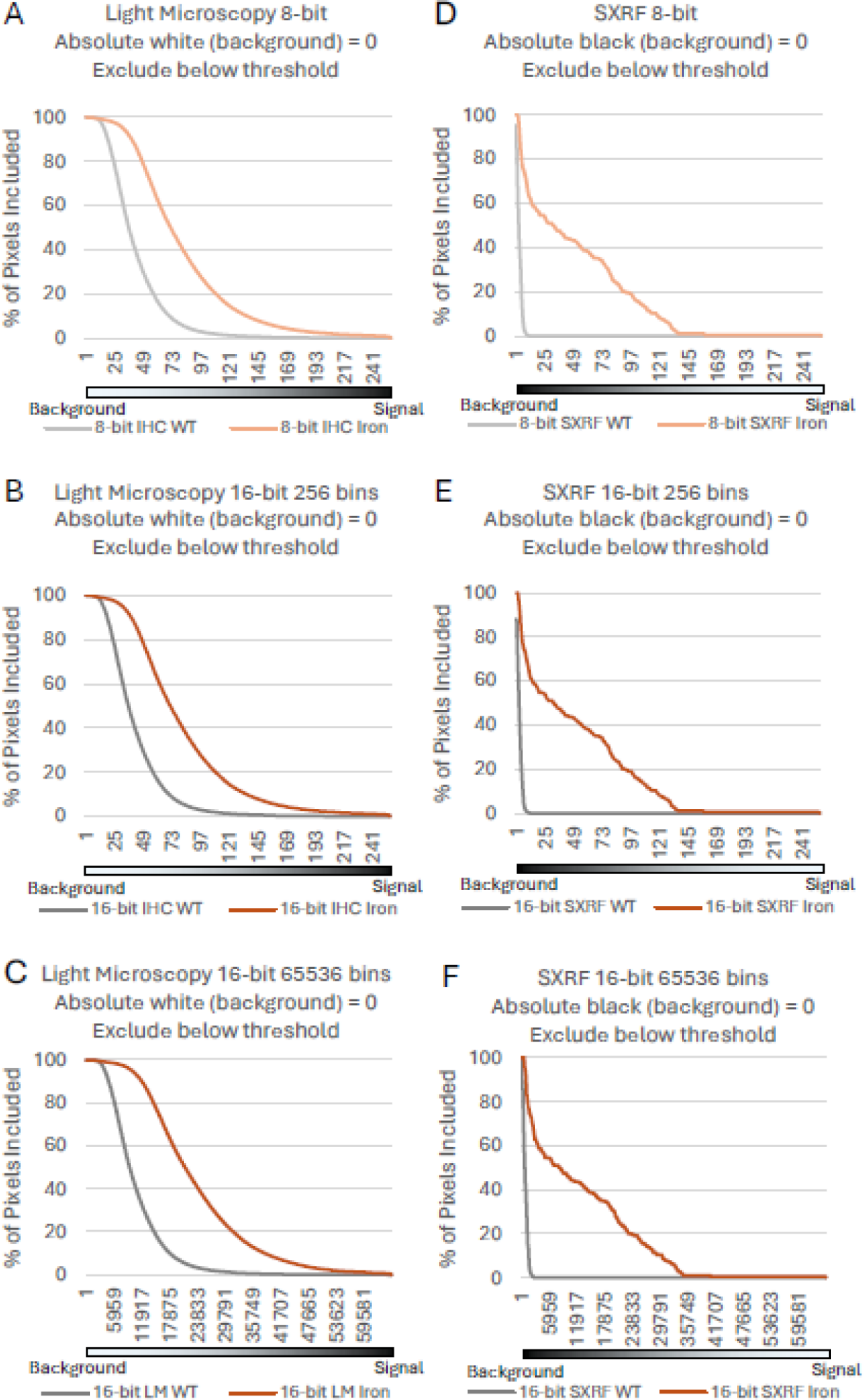
Cumulative threshold spectra for light microscopy (A, B, C) and SXRF (D, E, F) images of pixels retained after picking threshold values at each PI, with *X*-axis set with signal values higher than the background and pixels excluded below the threshold, i.e., correct setting and valid outcome. When 16-bit data are represented using 256 bins (ImageJ default), this compresses the data with information loss, and the resulting profiles are identical to those of 8-bit data (B vs A; E vs D). Selection of the option to represent 16-bit data using 65,536 bins provides more information and higher resolution compared to 8-bit data (C vs A; F vs D).

### ImageJ Default Directionality of *X-*axis Scale and Implications for Different Platforms

The ImageJ default is intensity 0 for absolute black pixels and larger intensities for pixels with lighter colors. This is appropriate for images obtained by platforms generating fluorescence-based outputs. Valid cumulative threshold curves for such images will be generated by using the ImageJ default setting and excluding pixels below the threshold (e.g., Figure 4A). It is important to recognize that this is not appropriate for microscopy images where the material of interest appears darker than the background. When a colored photomicrograph image is converted from RGB color to a grayscale format, the ImageJ default setting assigns lower intensity values to darker pixels. If not recognized, cumulative threshold analysis on images with signals darker than background may generate invalid results if pixels below the threshold are excluded (Figure 4B). This can be addressed by excluding pixels above the threshold instead (Figure 4C); however, this is counterintuitive and can make interpretation more difficult.

Alternatively, as described for converting RGB images to grayscale above, the *X*-axis for light microscopy images can be inverted so that the signal now corresponds to absolute black. Pixels can then be excluded below the threshold as usual (Figure 4D, our preferred option).

### Converting Images to Lower Bit Depth Formats and Recommended Range Settings

ImageJ converts 16-bit and 32-bit image data to 8-bit format by linear scaling of the absolute minimum and maximum pixel intensities to the range of 0 to 255 (ImageJ.net user guide).^21^ Users do not always realize that how data are converted to 8-bit from higher formats depends on whether “Scale When Converting” is selected (recommended default) or that the scale used depends on the *Brightness/Contrast* settings. If *Scale When Converting* is not selected in the *Conversions* settings within *Options* when converting images to lower bit formats, data are compressed by clipping or rounding without scaling, e.g., when converting 16- or 32-bit depths to 8-bits, the first 256 or 65,536 pixel intensity bins of these formats, each encoded by a unique 16- or 32-bit code, are all reduced to the same 8-bit binary code for the first bin of the 8-bit scale, starting at 0, representing a single pixel intensity.

With *Scale When Converting* selected (recommended default), ImageJ takes the image *Display Range* and linearly rescales to fit the new bit format range (e.g., 0–255, 0–65,535), before clipping or rounding based on *Minimum* and *Maximum* values entered under *Brightness/Contrast*. Each pixel previously within the *Display Range* is binned according to its relative position within the range and assigned the bin number as its new relative intensity value, i.e., the bin number at lower bit formats becomes a proxy for the relative intensity of the pixel within the new range. It is important when presenting pixel intensity information at lower bit formats to also provide the intensity ranges of the original data images to allow meaningful comparisons between images, particularly when comparing images from different experimental groups, such as low and high iron, if the true *Minimum* and *Maximum* values differ substantially between some of the images.

Any pixels below or above the image *Display Range* used for the conversion will be assigned, respectively, to 0 or to the maximum bin number in the format. As the information for these pixels outside of the *Display Range* will then be lost, making comparisons difficult, it is important to make sure that the image *Display Range* is correctly set for the image itself and for any other images in the groups being analyzed. As above, we recommend setting the image display and the *Histogram* analysis ranges using 0 and the maximum PI values in all images as the *Minimum* and *Maximum*.

This is particularly important when using images in the 32-bit format, which allows floating point scale PI values (Table 1, Introduction), and which does not have a fixed PI range, unlike 8- and 16-bit formats. For example, the SXRF images did not have the same *Display Range Minimum* and *Maximum* values, so they could not be accurately compared if then converted to a lower bit format because the default recommended *Conversion* settings linearly scale the min/max values of the *Display Range* disproportionately down to 0–65,535 or 0–255. Using the above strategy allows the images to be compared on the same nontransformed scale without disproportionate rescaling of the two different images.

### Issues Visualizing SXRF Images or Other Images at 32-Bits

The examples for the two different platforms used here generate very different curves (Figures 4, 5). This is not solely a reflection of the platforms *per se* but is partly due to the very small total pixel numbers in the particular SXRF image ROIs used here (Control 530, High Iron 194), which cover only a very tiny region of the brain (fourth ventricle). The SXRF images used also had different dimensions in pixels, resulting in differently sized display windows which, when expanded to similar sizes on the screen, were at different zooms and had different pixel sizes. It is not always obvious that this is occurring and can lead, at least initially, to inappropriate comparisons and interpretations. Appropriate normalization is discussed below.

The SXRF platform has the capacity to resolve a far greater number of intensities than 8- or 16-bit formats can represent; the 32-bit format uses 24 bits for digits and 8 bits for other information, as specified by the IEEE conventions (see Introduction), theoretically allowing differentiation of 2^24^ intensity values. However, as ImageJ is only able to display 256 different shades of gray, this resolution is not reflected in the image views provided by ImageJ. While beyond the scope of this paper to examine this in depth, it is again important to bear in mind the difficulties in visualizing and conceptualizing data at formats above 16-bits and potential pitfalls for subsequent analyses.

### Generation of Different Types of Histograms in ImageJ

ImageJ does not distinguish clearly between the histogram in the *Brightness/Contrast* dialogue box, which spans the full pixel value range and we will term the “*Brightness/Contrast* histogram”, and the histogram generated for data analysis using the *Histogram* function, which we will term the “analysis histogram”. After an image is imported into ImageJ, a range for image visualization needs to be specified using the setting in the *Brightness/Contrast* box. This is termed the “*Display Range*”. As a default, the *Display Range* usually has the darkest pixel value of the image as the minimum and the brightest pixel as the maximum, but the minimum and maximum can be changed in the *Brightness/Contrast* dialogue box by dragging the min/max sliders or typing in the desired values if these are outside the pixel value range. It is important to recognize that if changes are made, this can sometimes substantially alter the appearance of the image. ImageJ then recreates the image by visually representing the pixels within this display range by linearly mapping their intensity values on an 8-bit grayscale. If the minimum and maximum are set within the full pixel value range, all pixel values below the *Display Range Minimum* will be represented in the image as absolute black, and all values above the *Display Range Maximum* will be represented as absolute white.

A range also needs to be specified in the *Histogram* function for data analysis, which we will term the analysis range. In the *Histogram* dialogue box, selecting the *Use Pixel Value Range* option creates the analysis histogram using the full range of pixel values present in the image. When this option is not selected, the range used for the histogram is based on the *X min* and *X max* values set in the *Histogram* box. By default, ImageJ may set these values to be the same as those of the image display range used for visualization when working with a new image. Researchers may not realize that both visualization of the image and determination of the data range used for the analysis histogram are then dependent on the *Display Range*.

Alternatively, the researcher can select desired values for X min and X max, for example, to only include a restricted intensity range of the entire image, such as putative signal pixels. For all bit formats, if a restricted range is used for the histogram for one image in a set, then ImageJ analyzes the data only within that range for that image and possibly also for other images in the same set if the range values are not appropriately reset for each image.

### Recommended Range Settings When Analyzing Multiple Images

When analyzing more than one image, we recommend setting both the image *Display Range* and the analysis histogram range to have a minimum value of 0 and a maximum value that matches the highest PI value in the set of all data images used for analysis to ensure that all pixels in each ROI or image are being included in the data. Using a minimum value of 0 for all images prevents the images from being resized differently by having different minimum values stretched by different amounts to 0. Alternatively, depending on the data distribution, researchers may choose to exclude some pixels, for example, near the start or end of the range. It is important that this is done transparently and that the rationale and criteria used for exclusion are provided.

### Histograms

As illustrated above, analysis histograms were generated in the ImageJ program using 8- and 16-bit formats for both the histochemical and SXRF data images (Figure 1E–P) to compare differences in the raw data at each of the different bit formats. As explained in the Methods, while ImageJ histograms provide a convenient and informative visualization tool, they are only an approximation and can be misleading because the vertical axes are unlabeled, and the magnitude can change without any indication when the number of bins is changed.

### Effects of Different Bit Sizes and Bin Numbers on Distribution of Cumulative Threshold Data

The format for presenting 16-bit data (65,536 intensity values) can be managed by selecting the number of bins used via the Histogram function. It is important to realize that with the default setting of 256 bins, ImageJ linearly compresses the data onto a 0–255 scale, effectively losing information. This loss is not irreversible as the original data can still be retrieved by altering this setting, but if not recognized, it can affect subsequent analyses (Figure 5). Alternatively, the 16-bit data can be presented on a scale of 0–65,535 bins.

### Areal Density Plots

To address this, we also generated the areal density plots of the pixel numbers at each PI from the raw data represented in the histograms. We then investigated how these density plots varied for light microscopy (Figure 6) and SXRF (Figure 7) with different settings using 8- vs 16-bit formats.

**Figure 6:**
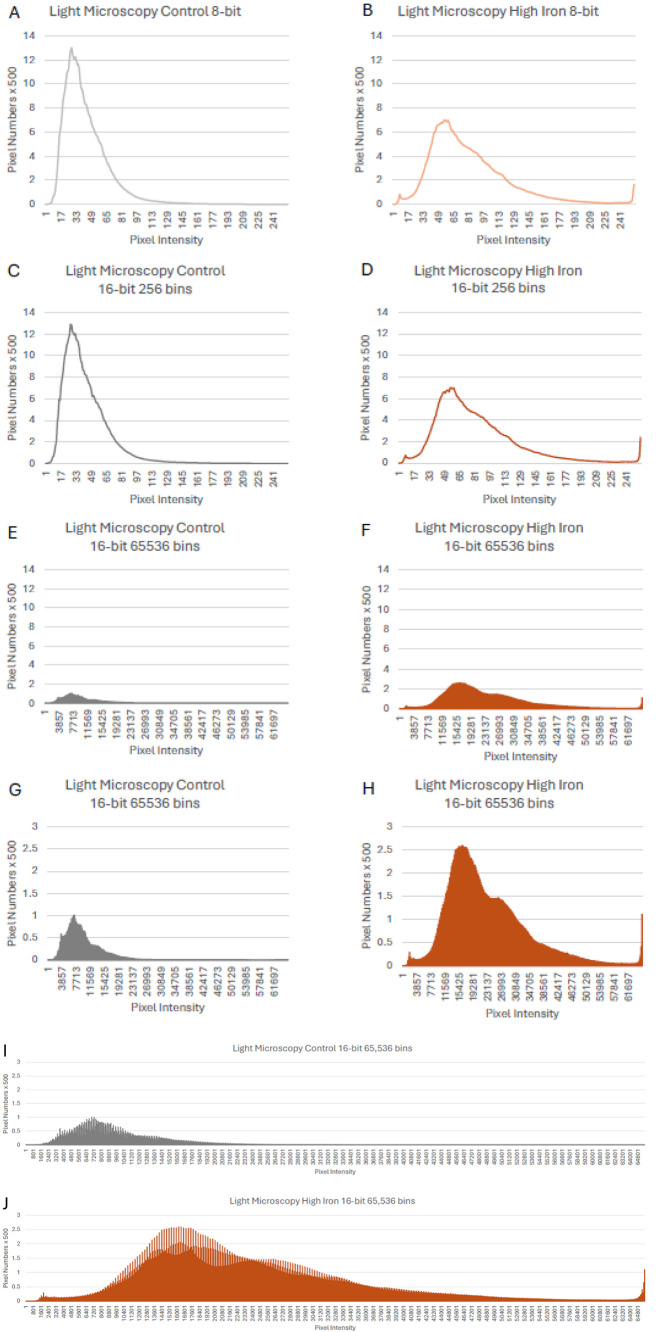
Areal density plots of control and High Iron light microscopy hemisphere images at different bit formats normalized by the total pixel numbers in each image ROI. (A, B) Pixel distribution of images at 8-bit depth. (C, D) Pixel distribution of images at 16-bit depth with the full range separated into 256 bins. (E, F) Pixel distribution of images at 16-bit depth across the full range of all 65,535 relative PIs. (G, H) The 16-bit full range distribution graphs with the *Y*-axes rescaled for comparisons with (E) and (F). (I, J) Plots in (G) and (H) displayed on expanded *X*-axes.

**Figure 7:**
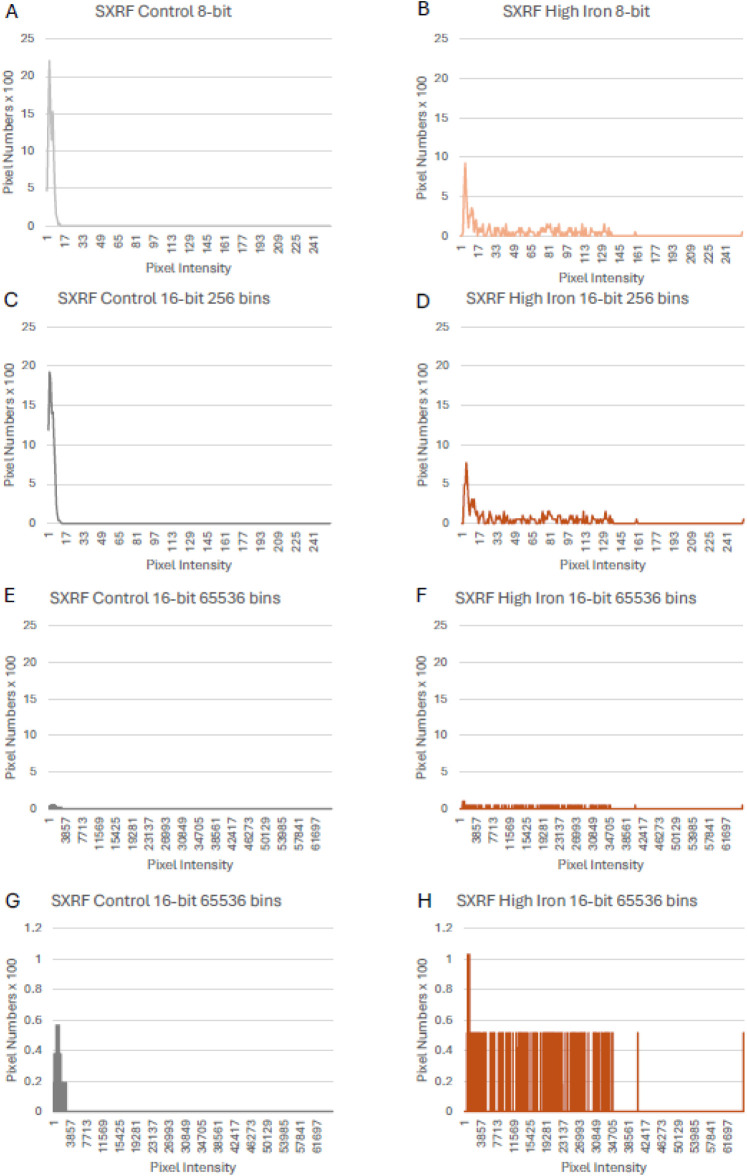
Areal density plots of control and high iron SXRF images at different bit formats normalized by the total pixel numbers in each image ROI. (A, B) Pixel distribution of images at 8-bit depth. (C, D) Pixel distribution of images at 16-bit depth with the full range separated into 256 bins. (E, F) Pixel distribution of images at 16-bit depth across the full range of all 65,535 relative PIs. (G, H) The 16-bit full range distribution graphs with the *Y*-axes rescaled for comparisons with (E) and (F).

As discussed above, data are lost if the ImageJ default of 256 bins is used when converting from 32-bit to 16-bit data. Instead, the data should be presented on a scale of 0–65,535 bins.

The foregoing highlights important considerations for preparing data for cumulative threshold analysis. This involves conversion to 32-bit grayscale (if needed), checking and inverting *X*-axis directionality (if needed), setting image *Display Range* minimum and maximum values, conversion to 16-bit format if not working at 32-bits, generating analysis histogram of the Image/ROI using 65,536 bins, inspecting for data anomalies, and copying the vetted analysis histogram data into Excel. We will now consider in more detail how the cumulative threshold analyses are performed in Excel.

### Exporting Data to Excel for Normalization, Areal Density Plots, and Cumulative Threshold Analysis

Analysis histogram data exported into Excel are first normalized by the total pixel count in each image. Normalizing is important because images of different sizes can vary considerably in pixel numbers, leading to invalid comparisons. As expected, the density distribution peak for the mice with elevated brain iron is shifted toward higher pixel intensities for both platforms and both bit formats (Figures 6, 7). For the 16-bit format using the 0–65,535 scale, the data are distributed across more bins, so the number of pixels in each bin is smaller (Figures 6E, F, 7E, F), and the data are also decompressed, so more detail is visible, e.g., more local peaks and troughs appear. This can be more clearly visualized using a different *Y*-axis scale (Figures 6G, H and 7G, H). This can provide important insight into the data that can vary depending on the platform and other factors, such as the total number of pixels in the ROI.

### Saturation and Compression of 32-Bit Data When Converting to Lower Bit Depths

The 32-bit format represents the intensity range of the data with greater radiometric resolution and accuracy than those allowed by 8- and 16-bit formats. When 32-bit data are converted to 16-bits or 8-bits, respectively, the number of data points of different intensities compressed into each individual bin increases from 1 to 65,536 or to ∼65,536 × 256 = 16,777,216, respectively. This is likely to involve information loss. Saturation may occur when a group of pixels spread across multiple bins representing different intensities within a particular intensity range at 32-bits are grouped into one bin representing a single intensity at lower bit formats. The actual intensity of each pixel within the compressed bin has not necessarily been reset to a single value in ImageJ but may still be retained in the ImageJ “memory”/data set. This was observed for SXRF images after conversion to lower bit formats and, to a lesser extent, for the light microscopy images, consistent with SXRF detecting a much larger range of energy intensities, allowing iron imaging within a wider concentration range.

### Normalization and Cumulative Threshold Analysis

We next applied the cumulative threshold method of Johnson and Walker,^3^ after first normalizing the raw pixel numbers to the total number of pixels in the ROI in each image (Methods). Normalization is essential for valid comparisons of data obtained from different images for studies where it is not feasible or appropriate to use ROIs of matched area. For example, brain size and sometimes other features can differ in unexpected ways between and within experimental groups, and there can sometimes be unavoidable sampling variability across the particular brain sections available for analysis. Random sampling of the ROIs of matched areas can be problematic. As we have reported previously,^22–25^ brain iron distribution has high regional and structural heterogeneity. This is generally less of a problem in the human brain, with dimensions of ∼17 cm long × 14 cm wide × 9 cm deep, where samples of ∼1 cm in dimensions may be selected for sectioning. In the mouse brain, which is only ∼1.4 cm long × 1 cm wide × 0.7 cm deep, even small variations in the precise locations of sections available for analysis can change profiles substantially, for example, due to inclusion of a tiny amount of choroid plexus, a bilayered ribbon of cells lining the cerebral ventricles that has extremely high iron content compared to the rest of the brain.

### Calculating Cumulative Thresholds

Cumulative threshold graphs (Figures 4, 5) were generated from the normalized pixel numbers by progressively setting the threshold at each PI level and plotting the percentages of pixels below that threshold. The following function was developed to calculate in Microsoft Excel the signal estimate at each threshold as the number of pixels retained in an image ROI after excluding everything below the threshold value:

$$\mathrm{Signal}=\frac{\left(\overset{255}{\underset{i=PI}{\sum }}n_{i-1}\right)-n_{PI-1}}{\left(\overset{255}{\underset{i=1}{\sum }}n_{i}\right)}$$

This function calculates, for each pixel intensity PI, the amount of signal at or above the threshold value by summing the pixel numbers at each pixel intensity from the previous PI (PI minus 1) to 255 and then removing the number of pixels at that previous PI. The resulting value is then normalized by the total number of pixels in the ROI, as detailed above, and converted to the percentage of signal retained at each intensity value. The Excel process for calculating signals at or above cumulative thresholds is given in Appendix 1.

### Differences between Selection of Signal at Different Thresholds

To investigate effects of selecting different thresholds on assessing entities of interest, we selected matched ROIs of cortical areas containing plaque structures of interest (Alzheimer’s related plaques) in each of the control and High Iron light microscopy hemisphere images and applied the same set of five thresholds used above (Methods) to both areas (Figure 8). For the control image, PI 75 was considered suitable for selecting most plaque structures stained for iron, while higher PIs failed to reliably identify some plaques. However, as the High Iron image is more strongly stained overall, consistent with higher iron content, for the High Iron image, almost the entire ROI was included at PI 75, whereas some plaque structures were not reliably detected at PI 125. This suggests that even if a threshold may be suitable for analyzing structures within one group of images, it may not be suitable in other groups. Fine tuning threshold selection between PI 75 and PI 100 may help resolve this problem. For grayscale using NTSC, analysis at 16-bits substantially affected the % difference compared to 8-bits but both gave similar significance outcomes (Figure 8B compared to 8C). For grayscale using RGB averages, both 8- and 16-bits had similar % differences and significances for all PIs investigated (Figure 8D, Figure 8E). Comparing the NTSC and RGB analyses at 8-bits, RGB averages had much lower % differences from PI 75 and above, with a loss of significance at PI 75. This highlights the potential inaccuracy of using the NTSC formula for data analysis, although this formula is more representative of how the human brain perceives images.

**Figure 8:**
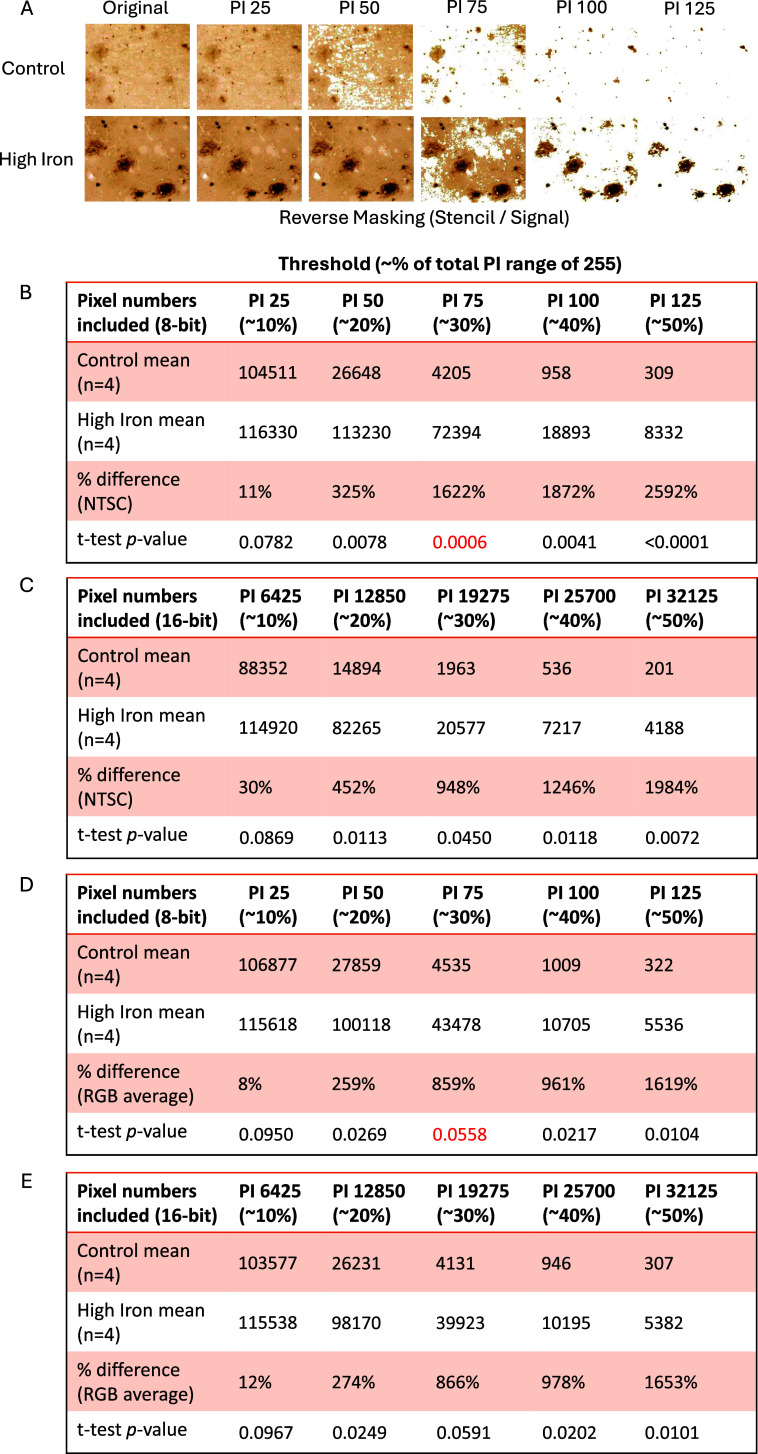
Effects of different thresholds on analyzing differences between groups. (A) Examples of stencil images for comparison of different thresholds. (B, C) Analyses comparing region-matched selections of the same size from each of the control and high iron light microscopy hemisphere images converted to grayscale using the NTSC formula (*n* = 4 regions/image) at 8-bits (B) and 16-bits (C). (D, E) Analyses comparing the same areas converted to grayscale by averaging the RGB channel values at 8-bits (D) and 16-bits (E).

### Selecting Different Thresholds for Different Experimental Aims

As mentioned above, a threshold appropriate for one image may not be appropriate for the other images used. This also applies to selecting thresholds for looking at different objects, for example, a lower threshold PI may be selected when wanting to select all fibrous structures staining for iron which are darker than the extracellular “background” or when wanting to select only plaques or iron-rich cells which have darker stain than the fibrous structures (Figure 9).

**Figure 9:**
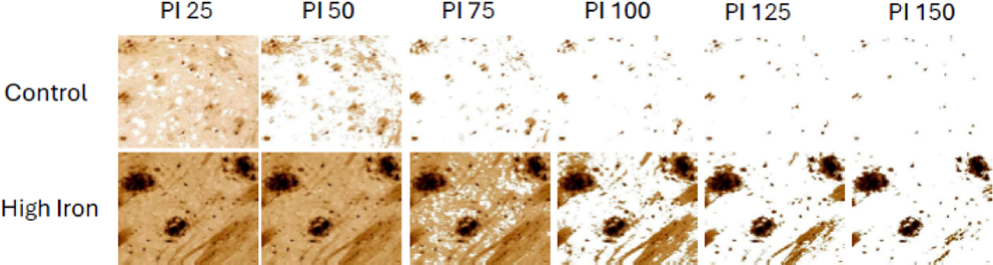
Stencil images for investigating how selecting thresholds to detect specific structures (fibrous, plaque) for one comparison group affects the amount of signal being detected on the other groups. Thresholds applied had PIs corresponding to approximately 10%, 20%, 30%, 40%, 50%, and 60% of the full intensity range at 8-bit.

### Summary and Recommended Guidelines

In summary, cumulative thresholding is a valuable strategy for image analysis, but it is important to be aware of the features and idiosyncrasies of programs being used. While 8-bit formats are likely to be appropriate for many purposes, researchers are recommended to check if using higher bit formats may affect the outcomes of the particular analysis being used before proceeding. Below, we provide some guidelines for avoiding pitfalls.

1. Select *Scale when converting* in *Conversions* option settings before modifying images.
2. Convert RGB images to grayscale with *Weighted RGB conversions* unselected (i.e., use the average of color channels) in the *Conversions* option.
3. Immediately after converting RGB images to grayscale, ensure *X*-axis directionality is appropriate for the platform before making further changes.
4. Before converting to lower bit formats or generating analysis histograms, set the *Brightness/Contrast* image display range min/max to 0 and the maximum intensity of the images in the data set being analyzed.
5. When generating analysis histograms, ensure settings autopopulated by ImageJ in the *Histogram* dialogue box are modified appropriately for the analysis.
6. All procedures and settings used for threshold analysis and quantification of images should be transparently reported, along with criteria for selection of specific threshold or threshold ranges.

## Discussion

### Summary and Significance

Our findings support the proposal that cumulative threshold approaches over an extended range of signal intensities can provide useful information for semiquantitative assessment of the areal densities of metal distribution images, exemplified here by iron. This is valuable for comparisons of iron staining intensities in targeted histochemically labeled structures. However, even the simple examples reported here demonstrate the need for caution in how cumulative threshold analyses are performed and interpreted. How this affects analyses of different data sets can vary depending on the imaging platform, samples, the type and purpose of the study, and the objectives of the study. Issues with saturation or resolution loss may lead to inaccurate interpretation.

### Advantages of Higher Bit Depth

Although the 8-bit format is widely used, we found that higher bit depth can sometimes give important insights into data distributions and anomalies not provided at 8-bits, particularly in reducing saturation effects at the high end of the intensity scale. However, the examples presented here show that even for data with clear separation of test and control groups within linear or other parts of the range used for analysis, it is important to recognize and avoid pitfalls that can include:

i. nontrivial information loss,
ii. digital, as opposed to modality-related, saturation, and
iii. different artifacts for different types of data both within and between modalities.

In general, for researchers working with ImageJ and Excel, routine use of 16-bit over 8-bit formats can provide increased resolution but may not significantly affect analysis outcomes, noting that 32-bit formats might provide more useful comparisons but may complicate or even invalidate analyses with programs unable to fully accommodate high bit formats.

While imaging approaches should facilitate objective assessment of map data, independent of modality, our findings showed that the form and extent of changes can differ substantially for both different imaging modalities and different image sets using the same modality. Here, we have shown examples of artifacts that can occur using images displaying different signal strengths (obtained from brains with high or low iron levels) measured by different modalities and/or differing in the pixel numbers sampled.

### Comparisons with Past Research

Traditional threshold setting usually does not apply objective selection of a cutoff point. Early solutions, such as Otsu’s method, assume bimodality of the data, i.e., clear separation of noise vs signal (sometimes referred to using terms such as background vs objects or foreground). This may not be suitable for examining histochemistry or immunofluorescent images that have a continuous spectrum.^9^ Johnson and Walker (2015) have improved on this by analyzing the full range of possible thresholds for their data set, recognizing that different thresholds need to be selected for different purposes and emphasizing the importance of transparent and well-justified rationales for selecting a particular threshold.^3^

Past literature has already discussed issues with traditional thresholding methods, and alternative analysis methods have been developed to address some of them. The “threshold gradient method” proposed by Frazier and Wang (2013) converts continuous pixel classification spectra into specific categories to analyze landscape structures across varying intensities. This is similar to identifying threshold ranges that show relevant differences in the data (Frazier and Wang, 2013), a concept applied by Johnson and Walker (2015) and considered above (Figure 5).

### Limitations

We have only analyzed a small number of sample images for only two different platforms, with the primary aim of illustrating conceptually (as opposed to empirically) some of the kinds of issues that can occur, without attempting an exhaustive catalog of the various problems that may arise for different platforms or for different kinds of images within a single platform. Also, we focused on ImageJ, which is freely available and widely used; however, although it is able to perform analyses using a 32-bit format, bin numbers available for analysis histograms may be restricted, affecting visualization. This is further limited by Excel, which only allows 2^20^ rows. Some of the issues identified, such as *X*-axis directionality, relate to ImageJ default settings and may not be issues for other programs, keeping in mind that, conversely, other programs may have different issues that need to be identified and managed transparently.

### Future Directions

We have identified some of the potential pitfalls in cumulative threshold analysis, and it will be important to explore these more in depth in further research. For example, it will be of value to validate semiquantitative analyses using this method against iron quantification methods that can incorporate standards to improve the representation of absolute values, such as Quantitative Susceptibility Mapping (QSM) of biometals by MRI. It will also be of interest to analyze images combining iron staining with other types of histochemical staining (e.g., hematoxylin/eosin or cresyl violet tissue staining) by using the ImageJ color deconvolution function to separate out the individual color channels and then analyzing the resulting images. Other possible approaches to improve the method may be including segmentation techniques in the image processing stage to increase the accuracy of the analysis, such as that developed by Abdolhoseni et al. (2019).^26^ To automate workflows and increase the accuracy of the analyses, strategies more appropriate for 32-bits and above need to be explored using MATLAB, which can support formats up to 64 bits or other high capacity software and alternative methodologies such as Python programming and machine learning techniques.

In conclusion, our findings support the recommendation of Johnson and Walker that researchers utilizing semiquantitative comparisons of digitized images should investigate, plot, and report the full details and effects of cumulative threshold selection and analysis strategies across the entire data range to facilitate assessment of the underlying rationales and validity of single- or multi-threshold selection strategies.

We have demonstrated that cumulative thresholding analyses can be performed with relative ease using ImageJ and Excel for people not using MATLAB, although care needs to be taken to accommodate the limitations and idiosyncrasies of different programs. It is important that more transparent, objective methods continue to be explored to improve the reliability of biometal analyses using such approaches. The strategies presented here may facilitate biometal analyses for research or clinical purposes by using a range of modalities.

## Supplementary Material



## Data Availability

Spectral data for Figure S1 can be accessed at http://wrap.warwick.ac.uk/196063/ and the other datasets generated during and/or analysed during the current study are available from the corresponding authors on reasonable request.

## Appendix 1

Excel steps for cumulative threshold calculation:

1. Insert the pixel count for each bin (i.e., data from the analysis histogram) into Excel.
2. Divide the pixel count in each bin by the total pixel count of the image or ROI (normalized pixel count in each bin).
3. Calculate the value of 1 (or 100%) minus the normalized pixel count for the first bin (first cumulative threshold value).
4. For each subsequent bin, calculate the cumulative threshold for that bin by excluding all pixels below that bin by subtracting the cumulative threshold of the preceding bin.
5. Repeat this for all remaining bins.

## Notes

Conceptual example showing comparison of peaks within SXRF spectra from two regions of interest (PDF)
