# Supplementary material for: Application of Cumulative Threshold Approaches to Tissue Iron Measurement on Histochemical or Synchrotron X‑ray Fluorescence Platforms
Source: Chem Biomed Imaging. 2026 Feb 16;4(7):1416–31. doi: 10.1021/cbmi.5c00230 (PMC13417511; doi:10.1021/cbmi.5c00230)
Supplement: Supplementary file 1 [file im5c00230_si_001.pdf]

Application of cumulative threshold approaches to tissue iron measurement on histochemical or synchrotron X-ray fluorescence platforms

Chan-An Lin [1*], Elvis Acquah [2], Jake Brooks [3], Joanna F. Collingwood [3], Daniel M. Johnstone [4], Adrienne E. Milward [1,5*], Rebecca J. Hood [4,6*]

1 School of Medical, Indigenous and Health Sciences, University of Wollongong, Wollongong, NSW, 2522, Australia
2 University of Utah, Department of Pathology, Salt Lake City, UT, 84108, USA
3 University of Warwick, School of Engineering, Coventry, England, CV4 7AL, UK
4 University of Newcastle, School of Biomedical Sciences and Pharmacy, Callaghan, NSW, 2308, Australia
5 The Florey Institute of Neuroscience and Mental Health, University of Melbourne, Parkville, VIC, 3052, Australia
6 University of Adelaide, School of Biomedicine, Adelaide, SA, 5005, Australia

Corresponding Authors: *Chan-An Lin cal875@uowmail.edu.au
* Adrienne (Lz) Milward amilward@uow.edu.au
*Rebecca J Hood Rebecca.hood@adelaide.edu.au

Table of Contents

Figure S1. Conceptual example for SXRF spectra from two regions of interest, showing the impact of threshold choice on SXRF map display.

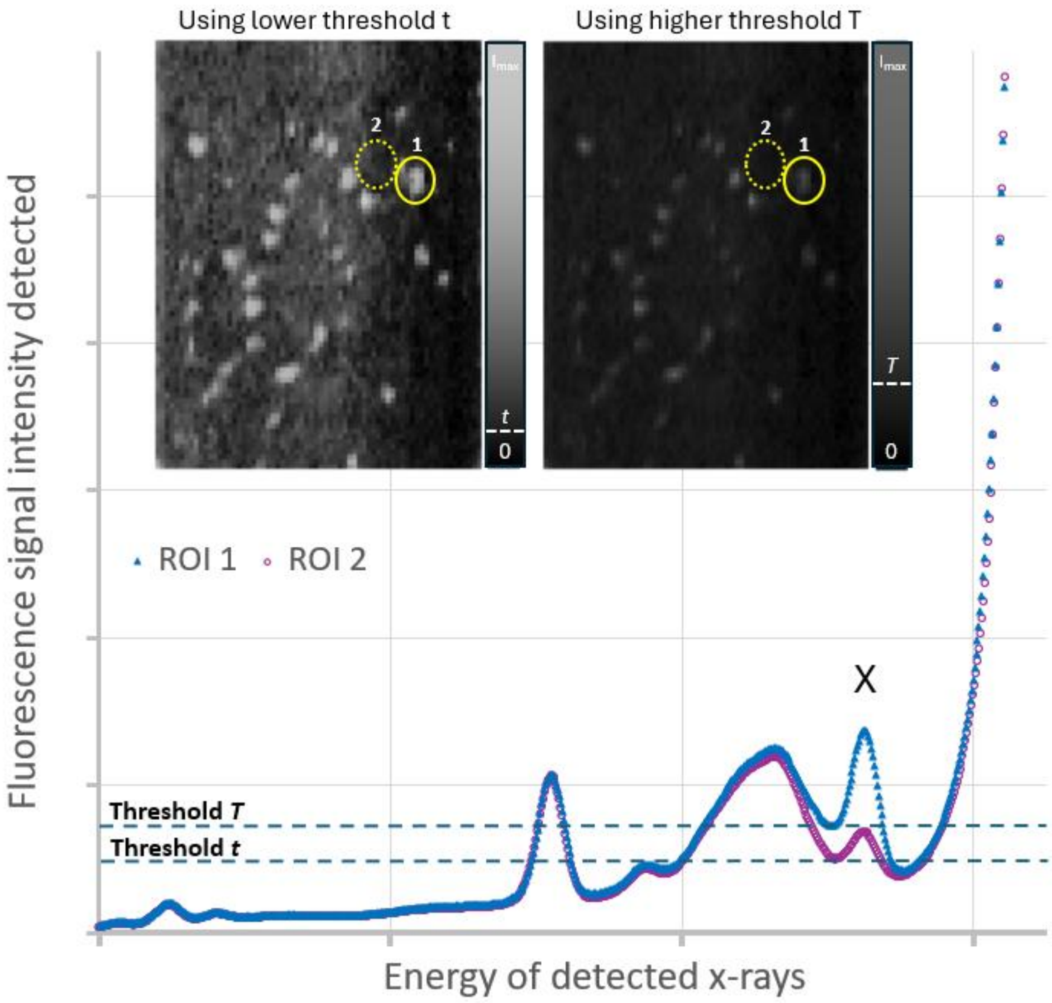

Figure S1. Conceptual example for SXRF spectra from two regions of interest, showing the impact of threshold choice on SXRF map display.

Illustrative SXRF spectra are shown for two regions in a tissue section. The positions of the peaks in the energy spectra and their amplitudes are defined by the chemical elements and their concentrations respectively. A full peak fit, including background subtraction, is performed for the spectrum associated with each pixel, to quantify the signal intensity for each detectable chemical element in that pixel. The resulting signal intensity matrix for each element can then be mapped as an image, as exemplified by the insets mapped from the signal intensity matrices for element X. As the signal peak for element X is higher in ROI 1 than in ROI 2, the pixels representing ROI 1 are mapped with higher intensity than those for ROI 2 in both images. The image on the left is mapped for a lower threshold *t*. The right image is mapped for a higher threshold *T*. This removes more signal so the image appears darker and the spectral values for element X that are mapped to ROI 2 are no longer above the threshold. (Example has been constructed to illustrate key concepts and does not represent real data).
